# Southern Medical College Commencement

**Published:** 1885-03-20

**Authors:** 


### SOUTHERN MEDICAL COLLEGE COMMENCEMENT.

The commencement exercises of the Southern Medical College took place at DeGive's Opera House, Atlanta, Ga., on the evening of March the 4th, 1885. The weather was rainy and inclement, and yet there was a very large audience in attendance, composed of the best citizens of the city.

The exercises were opened with prayer by the Rev. Dr. Mack, of Columbia Theological Seminary, after which Dr. W. P. Nicolson, the Dean, made his Annual Report, which showed the Institution to be in a highly prosperous condition.

The second course students of the present session numbered thirty-six, of whom the following gentlemen passed successful examinations, to-wit:

J. W. Anderson, Ga.
Oscar Attaway, Ga.
J. P. Ballenger, Ga.
E. V. Blount, N. C.
R. R. Bomar, Ga.
G. E. Camp, Ga.
B. L. Clifton, Ga.
W. J. Davie, Ky.
T. S. Few, S. C.
E. M. Fowler, Ga.
H. W. Guthrie, Ga.
J. H. Hall, Ga.
Charles Hamilton, Ga.
P. E. Hannah, Fla.
W. I. Hayes, Ala.
Jos. Hollingsworth, Ga.
D. B. Jackson, S. C.
S. M. Kimsey, Ga.
Nathan King, Ga.
R. M. McBee, Tenn.
W. Y. McClure, Ga.
M. T. Maxwell, Ga.
C. A. Moran, Ga.
J. E. Rogers, N. C.
W. P. Rushin, Ga.
W. B. Sanders, Ala.
J. W. Saunders, Ga.
G. W. Scroggs, Ga.
S. G. Smith, S. C.
J. W. Suggs, Jr., Ga.
J. O. Whatley, Ga.
H. C. Barnett, M.D., Texas, Ad Eundem Degree.

The above-named candidates were arranged in two divisions, on either side of the stage, and presented a fine array of intelligent and good-looking young gentlemen.

Between these, occupying the central and back part of the stage, were the following persons:

His Excellency, H. D. McDaniel, Governor of Georgia.

Judge Geo. Hillyer, Mayor of the City.

Rev. Dr. Mack, of Columbia Theological Seminary.

Rev. Jas. G. Armstrong, of St. Luke's Episcopal Church, Atlanta.

Also, the following members of the Faculty of the Southern Medical College:

T. S. Powell, M.D., President and Professor of Obstetrics.

R. C. Word, M.D., Vice President and Professor of Physiology.

J. F. Gaston, M.D., Professor of Theory and Practice of Surgery.

Geo. G. Crawford, M.D., Professor of Operative Surgery.

G. G. Roy, M.D., Professor of Materia Medica.

W. D. Bizzell, M.D., Professor of Theory and Practice of Medicine.

W. P. Nicolson, M.D., Dean and Professor of Anatomy.

A. G. Hobbs, M.D., Professor of Eye, Ear and Throat.

J. A. Burns, A.M., Professor of Chemistry.

W. S. Elkin, M.D., Lecturer on Minor Surgery.

F. H. Peck, M.D., Demonstrator.

The diplomas were next presented by the Dean, each candidate stepping forward as his name was called and receiving his diploma with a graceful bow, and then falling back into line. The first division having been thus served, the degree of Doctor of Medicine was conferred in appropriate terms, and in an impressive manner, by T. S. Powell, M.D., President of Faculty and Board. The same process was then repeated to the second division, after which President Powell addressed the graduates in a speech of great eloquence and beauty, Our space will not permit its publication here, but we may give it a place in a future issue.

The speech of the Valedictorian of the class was next in the order of exercises, and was an address ably written and well delivered by Dr. J W. Suggs, Jr., of Georgia.

The Annual Address by Rev. Jas. G. Armstrong was then made, and was characterized by marked and unusual ability, not only in the manner of its delivery, which was in that peculiar and impressive style for which Dr. Armstrong is noted, but in the deep thought and scientific research which was disclosed in the matter of the address. The subject of materialism was the interesting theme selected for the occasion, and the thoughts enunciated attracted the rapt attention of the audience during its delivery, of about one hour.

He stated that the Medical profession and that of the Ministry have an interest in common in the subject of materialism: the one approaches it from the physical, the other from the spiritual side. As to the ministry, its very existence as a profession depends upon the reality of the spirit.

If materialism be true, or if there be no spirit or soul in the man, then the doctor is only a veterinary surgeon, dealing with mere animals.

It is charged that the medical man is in danger of overlooking the reality of spirit existence, and is too much inclined to discuss the question as to whether the organism is the cause or the instrument of the spirit. Experiment seems to show that the removal of successive layers of the hemispheres of the brain reduces proportionately the operations of mind. If spirit is real, then the doctor's responsibility is far greater, as he may jeopardize both soul and body.

The consequences of materialism are of fearful importance.

Materialism robs us of the proof of a God, because it is only from a consciousness of our own existence that we can come to believe that there is any other existence. A man without a spirit could not perceive. Personality only can perceive personality, or have affiinity with personality. The instinct for personality, and for a personal God, shows itself in the lowest races of men, evidenced in sculpture, statuary, etc. Instinct is a proof of a personal God.

Another consequence of materialism is that it divests man of all responsibility for wrong-doing. If man is only an animal without an accountable spirit, and without a God, he, like the tornado that causes destruction in its path, is not to be blamed: the murderer of his wife and his children is to be pitied and not to be punished; the murderer must be turned loose upon society and government. Materialism is opposed to the best ends of law and government.

Materialism sustains freelovism by relieving man from his fears and his obligations to society and to God.

The tenets or mottoes of the materialist are specious and false—mere claptrap. One of them is that, "Thought is emitted by brain as music by the organ." In the case of music the antecedent and consequent can be shown: not so with thought. Tyndall himself says, in reference to the modus operandi of thought, that it is unthinkable. Another assertion of the materialist is that "Man is what he eats," and it is claimed that brain food, or phosphorus, furnishes material for thought; but facts have shown that brain development is the result of thinking.

Materialism contradicts the testimony of consciousness. It has been said that "Our sensation is all we know;" but we are conscious of more than sensation. We are conscious of thoughts and purposes. Consciousness of thought is as evident as of sensation. Sensation is from without: thinking and purposing is from within. We are conscious of a self-acting power beyond the sensorium.

The physician, next to the minister, ought to realize the vast worth of man as a spiritual being, and the doctor, next to the minister, ought to be the most spiritually minded of men, etc.

We have only mentioned imperfectly some of the thoughts contained in Dr. Armstrong's able address.

The closing part of the exercises was the delivery of the prizes. The Dean, as representative of the Faculty, delivered the first and second honors for highest proficiency in all the branches.

The first honor, a beautiful gold medal, was given to Dr. T, S. Few, of South Carolina.

The second honor was conferred upon Dr. J. H. Hall, of Georgia.

The prizes in the special branches were next bestowed, each professor calling out the recipient and delivering to him the prize, with appropriate remarks. The following are the names of the parties thus honored:

In Eye, Ear and Throat, an ophthalmoscope to Dr. J. H. Hall, of Georgia., by Prof. A. G. Hobbs.

In Practice, a gold medal to Dr. T. S. Few, of South Carolina, by Prof. W. D, Bizzell.

In Materia Medica, a case of instruments to Dr. W. B. Sanders, of Alabama, by Prof. G. G. Roy.

In Chemistry, a test apparatus to Dr. M. T. Maxwell, of Georgia, by Prof. J. A. Burns.

In Physiology, a pocket drug case to Dr. W. P. Rushin, of Georgia, by Prof. R. C. Word.

In Obstetrics, a gold medal to Dr. J. H. Hall, of Georgia, by Prof. T. S. Powell.

In surgery, a case of pocket instruments to Dr. J. H. Hall, of Georgia, by Prof. J. McF. Gaston

In Anatomy, a case of pocket instruments to Dr. T. S. Few, of South Carolina, by Dr. W. P. Nicolson.

A voluntary prize, by Mr. Joseph Jacobs, Druggist, of Atlanta, consisting of an order for an outfit of medicines, was bestowed upon Dr. S. G. Smith, of South Carolina, for his proficiency in pharmacy.

The several parts of the programme were interspersed with most delightfu music, many floral gifts were distributed by the ladies to the graduates and speakers, and the entire occasion was one of great attraction and interest.

With each successive year our citizens and the Profession are more and more impressed with the management of the Southern Medical College. The excellent facilities afforded by the Institution, its clinical and hospital advantages, the ability and energy of the Faculty, the thoroughness of instruction and the high moral and educational standard which it has taken, all commend it to the good will of the Profession and patronage of the people.

